# Pituitary Adenylate Cyclase Activating Polypeptide (PACAP) Pathway Is Induced by Mechanical Load and Reduces the Activity of Hedgehog Signaling in Chondrogenic Micromass Cell Cultures

**DOI:** 10.3390/ijms160817344

**Published:** 2015-07-29

**Authors:** Tamás Juhász, Eszter Szentléleky, Csilla Szűcs Somogyi, Roland Takács, Nóra Dobrosi, Máté Engler, Andrea Tamás, Dóra Reglődi, Róza Zákány

**Affiliations:** 1 Department of Anatomy, Histology and Embryology, University of Debrecen, Medical and Health Science Centre, Nagyerdei krt. 98, H-4032 Debrecen, Hungary; E-Mails: valaki006@gmail.com (E.S.); somogyics@anat.med.unideb.hu (C.S.S.); takacs.roland@med.unideb.hu (R.T.); luysica@gmail.com (N.D.); engler.mate.janos@med.unideb.hu (M.E.); roza@anat.med.unideb.hu (R.Z.); 2 Department of Anatomy, MTA-PTE “Lendület” PACAP Research Team, University of Pécs, Medical School, Szigeti út 12, H-7624 Pécs, Hungary; E-Mails: andreatamassz@gmail.com (A.T.); dora.reglodi@aok.pte.hu (D.R.)

**Keywords:** mechanotransduction, chondrocyte differentiation, Sonic hedgehog, Indian hedgehog, hypertrophy

## Abstract

Pituitary adenylate cyclase activating polypeptide (PACAP) is a neurohormone exerting protective function during various stress conditions either in mature or developing tissues. Previously we proved the presence of PACAP signaling elements in chicken limb bud-derived chondrogenic cells in micromass cell cultures. Since no data can be found if PACAP signaling is playing any role during mechanical stress in any tissues, we aimed to investigate its contribution in mechanotransduction during chondrogenesis. Expressions of the mRNAs of PACAP and its major receptor, PAC1 increased, while that of other receptors, VPAC1, VPAC2 decreased upon mechanical stimulus. Mechanical load enhanced the expression of collagen type X, a marker of hypertrophic differentiation of chondrocytes and PACAP addition attenuated this elevation. Moreover, exogenous PACAP also prevented the mechanical load evoked activation of hedgehog signaling: protein levels of Sonic and Indian Hedgehogs and Gli1 transcription factor were lowered while expressions of Gli2 and Gli3 were elevated by PACAP application during mechanical load. Our results suggest that mechanical load activates PACAP signaling and exogenous PACAP acts against the hypertrophy inducing effect of mechanical load.

## 1. Introduction

Cells of skeletal tissues such as bone and articular cartilage change their developmental program, normal life cycle and expression of extracellular matrix components upon mechanical stimulation. Mechanical load plays a crucial role in the development and regeneration of the articular cartilage [1,2,3] suggesting the presence of cellular mechanisms of mechanotransduction either in developing or adult chondrocytes. Various cell surface receptors can serve for mechanosensation of chondrocytes, such as certain purinoreceptors [4], integrins [5], stretch activated Ca^2+^ channels [6], TRPV4 [7] and NMDA receptors [8]. Downstream targets of these receptors can regulate the morphology, metabolism, proliferation and Ca^2+^ homeostasis of chondrocytes. Our group demonstrated a PKA regulated mechanosensitive activation of Sox9 and PP2A [9] and the involvement of the hedgehog signaling in mechanotransduction has also been proven by Wu and colleagues [10].

Pituitary adenylate cyclase activating peptide (PACAP) is one of the members of VIP-Secretin-GHRH-Glucagon superfamily and has been extracted from ovine hypothalamus over 25 years ago [11]. After describing the neuropeptide in the regulation of development and normal function of central nervous system, more and more peripheral tissues were demonstrated to express and be influenced by this neurohormone [12,13,14]. PACAP is a short peptide existing in two biological active forms; PACAP 1–38 and PACAP 1–27 [11]. Both forms have a very short life span [15]. Three main G-protein coupled receptors of PACAP have been identified; PAC1, VPAC1 and VPAC2, the latter two have lower affinity to the neuropeptide [16]. Activation of these receptors can regulate divers signaling pathways [17,18,19,20,21], from which the canonical signaling connection induces the activation of PKA and/or MAPK system [19,21]. The expression of PACAP and its receptors in chondrogenic differentiation has been demonstrated by our group [22]. We have also shown that exogenous addition of PACAP exerts positive effects both on chondro- and osteogenesis [22,23]. Multifactorial or pleiotrophic effects of the neuropeptide have been observed in various biological processes, and PACAP was proven to prevent apoptosis, ischemic conditions, and oxidative stress [24,25,26,27,28]. We have demonstrated that addition of PACAP rescued the cartilage formations during oxidative stress in a high density cell culture model [22] and antiinflammatory functions of the neuropeptide were found in other skeletal elements [26,29,30].

Chondrifying micromass cell cultures (HDC), established from cells of chicken limb buds of four-day-old chicken embryos, represent a well reproducible experimental model of cartilage formation [22,31]. In these cell cultures, chondrogenic cells differentiate spontaneously to chondrocytes on days two and three and a considerable amount of hyaline cartilage is produced by day six of culturing. Signal transduction processes governing consecutive steps of chondrogenesis can easily be followed or modulated externally in this system. Responsiveness of chondrogenic cells to uniaxial cyclic compressive load generated by our custom-made mechanical stimulator was demonstrated by the observation that this intervention lead to the increase of cartilage differentiation and modulation of PKA regulated signaling pathways [9].

On the basis of this PKA-dependence of the mechanotransduction in this culture, we hypothesized the role of PACAP signaling in this process. Therefore, the major goal of the present study was to clarify the role of PACAP-signaling in mechanosensitivity of chicken chondrogenic cells. We present evidence that expression of PACAP and PAC1 receptor is elevated by mechanical load. We prove that mechanical stimulus enhances the hedgehog signaling, while exogenous PACAP attenuates the activity of this pathway either with or without mechanical load.

Our results suggest that PACAP reduces the tendency of hypertrophic transformation of chondrogenic cells evoked by mechanical load and it implies that PACAP signaling may take part in the regulation of terminal differentiation of chondrocytes *in vivo*.

## 2. Results

### 2.1. Mechanical Stimulation and PACAP (Pituitary Adenylate Cyclase Activating Polypeptide) Administration Enhance Collagen Production

**Figure 1:**
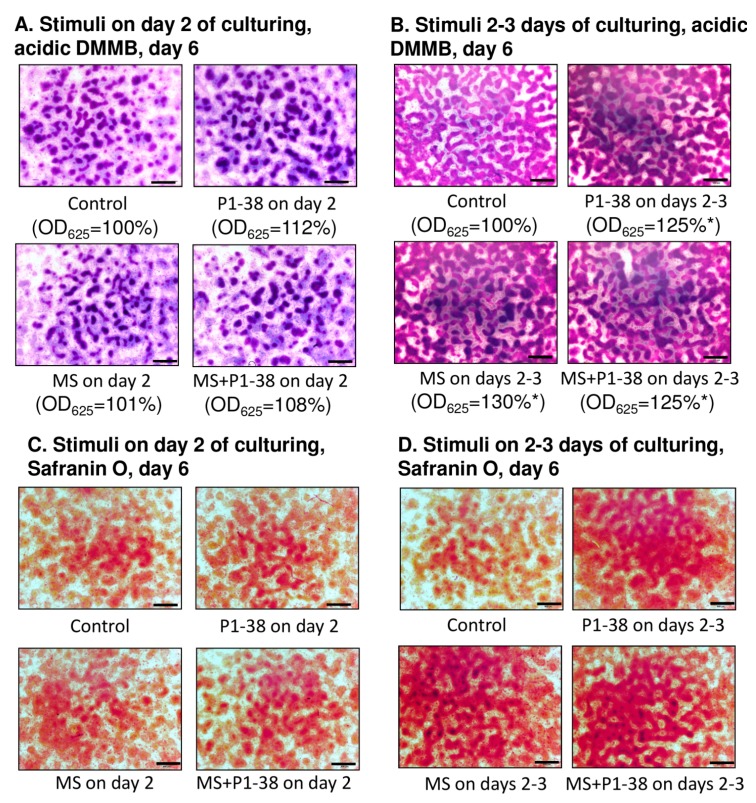
Effects of PACAP and/or mechanical load on matrix production of HDC. PACAP 1–38 at 100 nM was applied on day two of culturing (**A**,**C**) and continuously from day one (**B**,**D**). MS, mechanical stimulation applied on day two (**A**,**C**) and days 2–3 (**B**,**D**) for 30 min. (**A**,**B**) Metachromatic cartilage areas in six-day-old cultures were visualized with DMMB dissolved in 3% acetic acid. Metachromatic (purple) structures represent cartilaginous nodules formed by many cells and cartilage matrix rich in sulphated glycosaminoglycans (GAGs) and proteoglycans (PGs). Optical density (OD_625_) was determined in samples containing TB extracted with 8% HCl dissolved in absolute ethanol. Statistically significant difference of the extracted TB in cultures that received the loading regime and/or PACAP 1–38 *vs.* control cultures is marked by asterisk (* *p* < 0.05); (**C**,**D**) Safranin O staining for visualization of cartilage nodules of HDC. Original magnification was 4×. Scale bar, 500 µm. Representative data of three independent experiments are shown. (P1–38, PACAP 1–38; MS; mechanical stimulus).

Administration of PACAP 1–38 and/or application of mechanical load only on day-2 of culturing did not significantly alter the matrix production of differentiating chondrocytes (Figure 1A,C). In line with our previously reported results, either intermittent load [9] or addition of PACAP [22] significantly elevated the amount of metachromatic cartilage matrix produced in chicken micromass cultures by day six as it was revealed by dimethylmethylene blue (DMMB) and toluidine blue (TB) stainings (Figure 1B). We were able to verify these data with Safranin O staining as well (Figure 1D). Interestingly the combined application of mechanical load and PACAP did not have additive effect, the production of metachromatic cartilage nodules remained at a level similar to that of a single intervention (125% of control cultures; Figure 1A,D).

**Figure 2:**
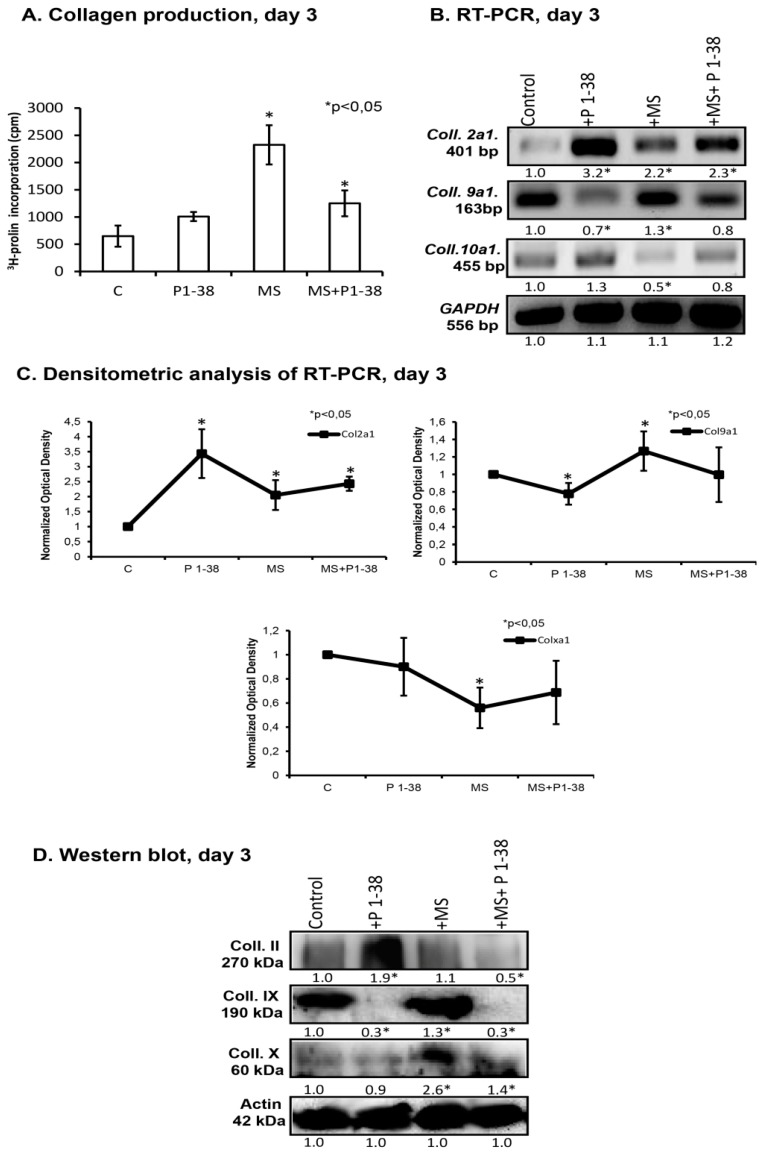

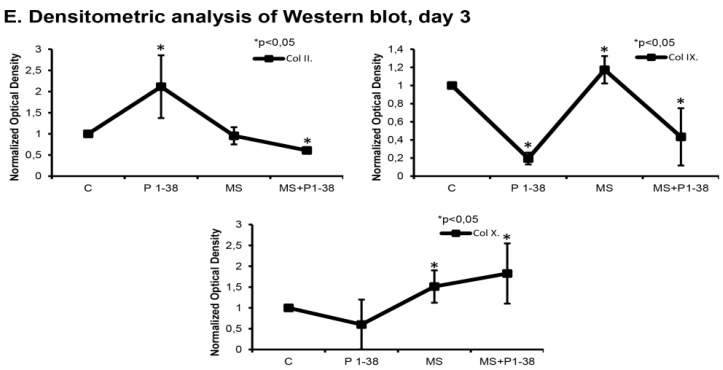
PACAP and/or mechanical load altered the collagen production of HDC. PACAP 1–38 at 100 nM was applied continuously from day one. Short time, transient MS, was applied on days two and three for 30 min on both days. (**A**) Collagen production of HDC was determined by ^3^H-collagen incorporation; (**B**) RNA expression of collagen type II (*Col2a1*), collagen type IX (*Col9a1*) and collagen type X (*Col10a1*) on day three. *GAPDH* was used as a control; (**D**) Protein expression of collagen type II, collagen type IX, collagen type X on day three. Actin was used as a control. For panels (**B**,**D**) numbers below signals represent integrated densities of signals determined by ImageJ software. Representative data of three independent experiments are shown; (**C**,**E**) Statistical analysis of RT-PCR and Western blot data. All data presented are the averages of at least three different experiments. Statistical analysis was performed by Student’s *t*-test. All data were normalized on GAPDH/Actin and data are expressed as mean ± SEM. (C, control; P1–38, PACAP 1–38; MS; mechanical stimulus). Statistically significant difference between collagen production rate of cells in cultures that received the loading regime and/or PACAP 1–38 *vs.* control cultures is marked by asterisk (* *p* < 0.05) in all panels.

Collagen fibers play an essential role in the establishment and maintenance of proper biomechanics of cartilage. We followed up the influence of mechanical load and PACAP addition on collagen synthesis with the measurement of the incorporation of ^3^H-proline into micromass cultures. Mechanical load alone caused an almost threefold elevation of ^3^H-proline-incorporation, while application of PACAP alone did not cause any significant change of this parameter (Figure 2A). When PACAP 1–38 was administrated during mechanical load, lower ^3^H-proline-incorporation was detected in comparison to that of measured during mechanical load alone but still an elevation was demonstrated comparing with the untreated control (Figure 2A). Next, we monitored the mRNA and protein expression of various types of collagens, specific to cartilage, during mechanical load and PACAP administration. Upon mechanical stimulation, the mRNA expression of collagen type II (*Col2a1*) and collagen type IX (*Col9a1*) increased (Figure 2B,C), while collagen type X (*Col10a1*) responded with a significant decrease*.* PACAP 1–38 treatment also enhanced the mRNA expression of collagen type II (*Col2a1*) but it decreased the mRNA expression of collagen type IX, while type X collagen mRNA expression did not show any significant change (Figure 2B,C). Similarly to the results observed in relation to the metachromatic cartilage matrix, the presence of PACAP 1–38 during mechanical stimulation slightly attenuated the effects of mechanical load on the expression all of the investigated collagens (Figure 2D,E). Regarding the changes of the collagens at protein level, previously we demonstrated that collagen type II production was elevated during PACAP addition [22]. Mechanical stimulation did not elevate the collagen type II protein level, while the combined application of PACAP and mechanical load caused a significant decrease of it (Figure 2D,E). PACAP 1–38 significantly reduced collagen type IX protein level (Figure 2D,E). On the contrary, mechanical load caused a significant elevation of this collagen type, but it did not compensate the reducing effect of the neuropeptide during the combined application (Figure 2D,E). In contrast to the mRNA expression profile, treatment with PACAP 1–38 did not influence the amount of collagen type X protein, while the mechanical stimulus increased it (Figure 2D,E). Combination of PACAP and mechanical load resulted in an attenuated but still significant elevation of collagen type X protein level (Figure 2D,E).

### 2.2. PACAP and PAC1 Receptor Expressions Are Elevated by Mechanical Loading

**Figure 3:**
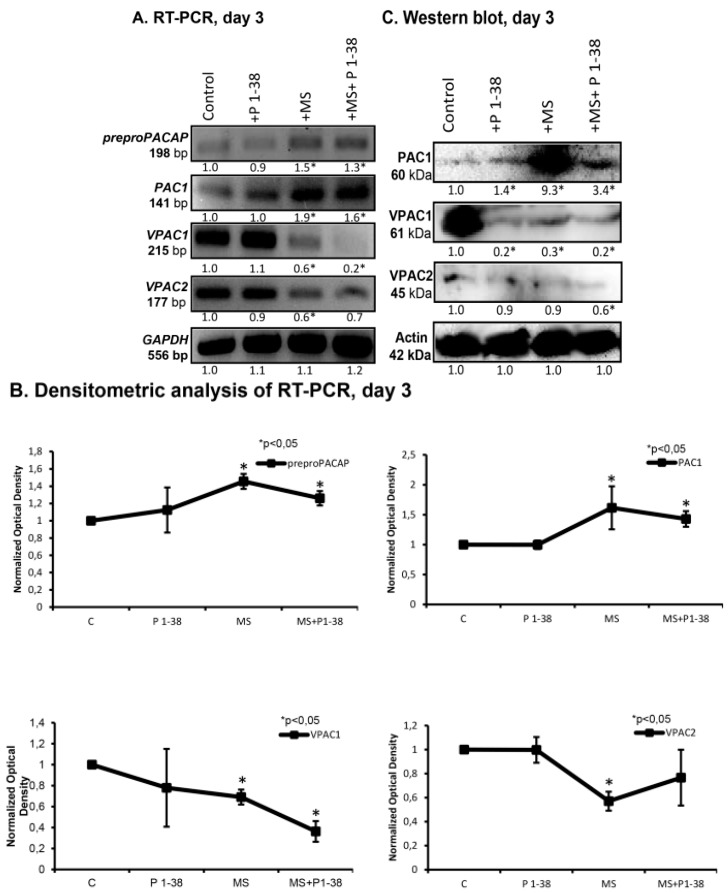

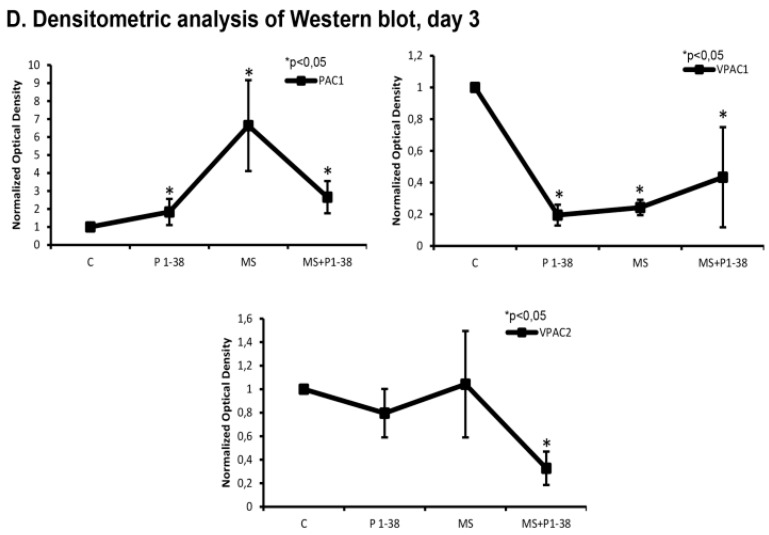
Expression of preproPACAP and PACAP receptors after PACAP administration and/or mechanical load in chondrifying micromass cultures. For RT-PCR (**A**) reactions GAPDH and for Western blot (**C**) reactions Actin was used as control. Optical density of signals was measured and results were normalised to the optical density of controls. For panels (**A**,**C**) numbers below signals represent integrated densities of signals determined by ImageJ software. Representative data of three independent experiments; (**B**,**D**) Statistical analysis of RT-PCR and Western blot data. All data are the average of at least three different experiments. Statistical analysis was performed by Student’s *t*-test. All data were normalized on GAPDH/Actin and data are expressed as mean ± SEM. Asterisks indicate significant (* *p* < 0.05) alteration of expression as compared to the respective control in all panels (C, control; P1–38, PACAP 1–38; MS; mechanical stimulus).

In the light of the general trophic effect of PACAP it could be a question of interest whether the neuropeptide plays a role in the cellular stress management of chondrocytes upon mechanical stimulus. Therefore, we investigated the expression of PACAP and its receptors following the application of mechanical load. The mRNA expression of preproPACAP was increased during mechanical stimulation and a modest decrease was detected at the presence of exogenous PACAP during the stimulation process (Figure 3A,B). The mRNA and the protein expression of PAC1 receptor was also elevated by mechanical load which effect was partly compensated by the PACAP administration (Figure 3A–D). When PACAP was added to the medium of cell cultures without mechanical load, it did not cause any significant alterations in the above expression levels (Figure 3A,B). We detected mRNA and protein expression of both VPAC1 and VPAC2 receptors in high density cultures from which only the protein expression of VPAC1 decreased significantly during the administration of PACAP 1–38 and mechanical load exerted the same effect (Figure 3C,D). Combination of the two interventions did not result in any change of these lowered expressions of VPAC1 (Figure 3A–D). VPAC2 receptor appeared with a low protein expression profile which further decreased during mechanical load and the combined treatments (Figure 3C,D).

### 2.3. Hedgehog Signaling Pathways Become Upregulated upon Mechanical Load, While PACAP Attenuates the Activity of This Pathway

**Figure 4:**
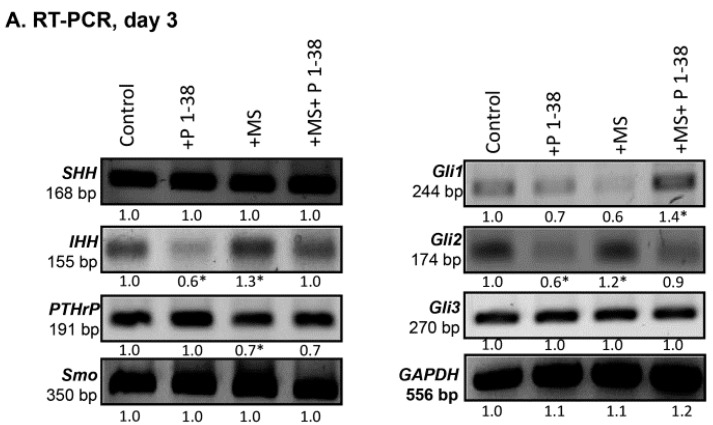

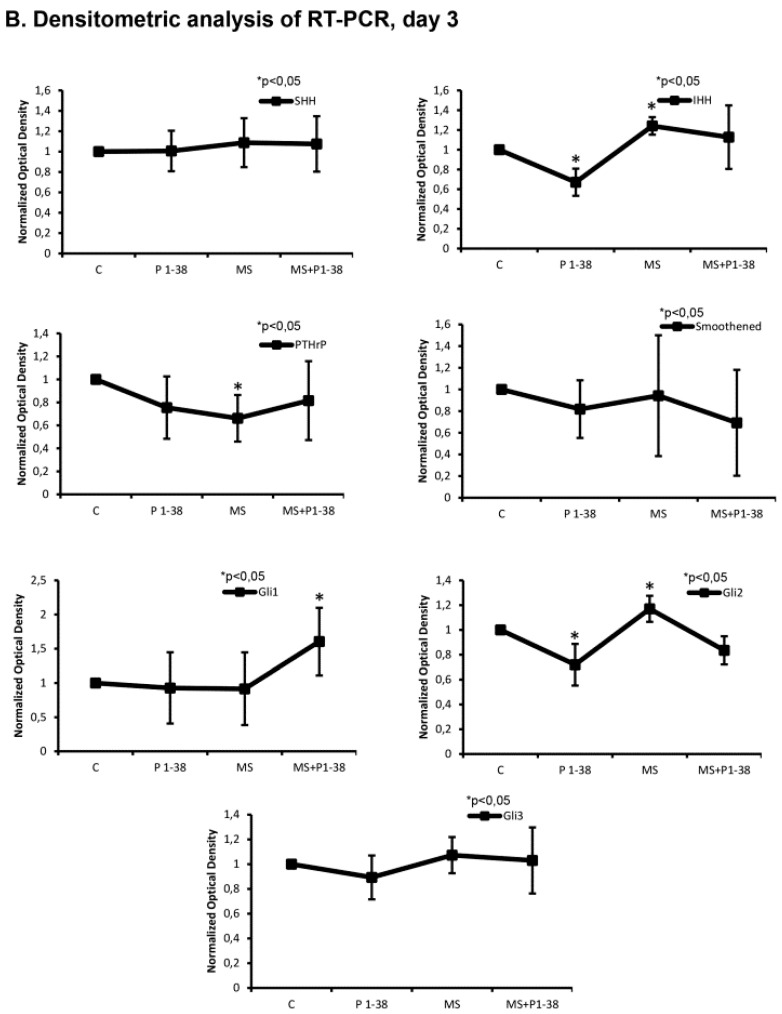

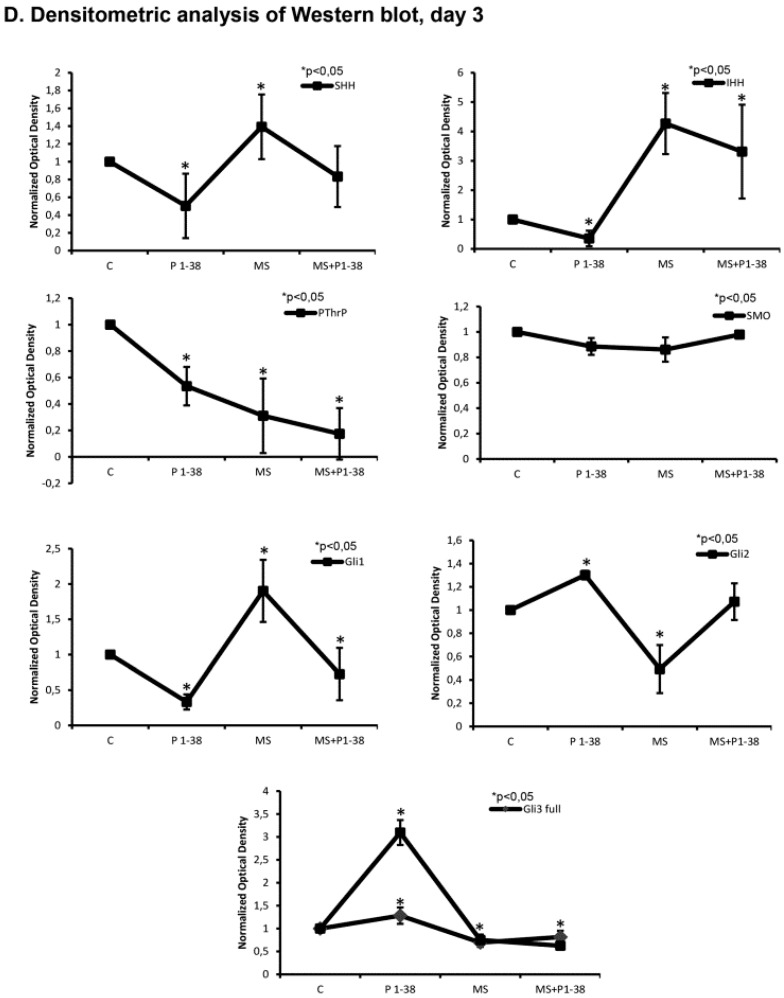
Effects of PACAP and/or mechanical stimulus on HH signaling of chondrofying cells. mRNA (**A**) and protein (**C**) expression of IHH, PTHrP, SHH, Smoothened (Smo), Gli1, Gli2 and Gli3 in HDC cultures on day three of culturing. For RT-PCR (**A**) reactions *GAPDH* and for Western blot (**C**) reactions Actin was used as control. Optical density of signals was measured and results were normalized to the optical density of controls. For panels (**A**,**C**) numbers below signals represent integrated densities of signals determined by ImageJ software. Representative results of three independent experiments are shown; (**B**,**D**) Statistical analysis of RT-PCR and Western blot data. All data are the average of at least three different experiments. Asterisks indicate significant (* *p* < 0.05) alteration of expression as compared to the respective control in all panels. Statistical analysis was performed by Student’s *t*-test. All data were normalized on GAPDH/Actin and data are expressed as mean ± SEM. (C, control; P1–38, PACAP 1–38; MS; mechanical stimulus).

The enhanced protein levels of collagen type IX and X imply that chondrogenic cells are shifted toward a prehypertrophic differentiation. As PAC1 receptor activation is known modulating hedgehog (Hh) signaling [32], we investigated the expression profile of this pathway. Indeed, we found a complex alteration of the expression of the members of Hh signaling in our experiments. Although the mRNA expression of Sonic hedgehog (*SHH*) was not altered by any of the applied treatments (Figure 4A,B), the SHH protein became almost undetectable upon PACAP 1–38 administration (Figure 4C,D). On the contrary, the mechanical stimulation resulted in a significant elevation of SHH protein expression which was normalized by the application of exogenous PACAP (Figure 4C,D). The mRNA expression of Indian hedgehog (*IHH*) was decreased, moreover, the protein expression reduced to an almost undetectable level after PACAP administration (Figure 4A–D). In contrast with this, the mechanical load enhanced the mRNA and protein expression of IHH (Figure 4A–D). Similarly to the SHH both the mRNA and protein expression of IHH were reduced by the combined treatment (Figure 4A–D). Expression of PTHrP, a signaling molecule known to work hand-in-hand with IHH, was decreased significantly during mechanical stimulation, while we failed to detect any alteration in its expression caused by PACAP 1–38 addition (Figure 4A–D). Combined administration of the neuropeptide with the mechanical load prevented the reduction of PTHrP expression (Figure 4A–D). Smoothened, the co-activator of hedgehog receptors did not show any mRNA or protein expression alteration upon any of the treatments (Figure 4A–D). Expression pattern of the downstream transcription factors of hedgehog signaling pathways such as Gli1, 2 and 3 were also monitored. The mRNA expression of *Gli1* was not altered upon either PACAP treatment or mechanical stimulation although the combined application elevated its mRNA expression (Figure 4A,B). The protein expression of Gli1 during PACAP addition reduced (Figure 4C,D) but significant elevation was detected after mechanical load (Figure 4C,D). When the mechanical stimulation was applied parallel with PACAP addition it significantly elevated the mRNA of Gli1 although its protein expression remained at a level similar to that of detected when PACAP was applied alone (Figure 4A–D). The mRNA expressions of Gli3 and Gli2, the transcription factors which can act as repressors of IHH or special localization is needed for their activation [33,34], were not altered under the effect of PACAP and/or mechanical stimulation (Figure 4A,B). Although protein expressions of both transcription factors elevated in the presence of PACAP 1–38 and reduced by mechanical load, they were not significantly altered during the combined treatment (Figure 4C,D). The shorter repressor form of Gli3 protein (*i.e.*, 83 kD) showed a prominent increase during PACAP administration but a significant reduction was detected during mechanical load (Figure 4C,D).

## 3. Discussion

Proper fetal limb development requires the physical forces exerted by the movements of the embryo [3]. Lack of the physical activity of embryos *in utero* or *in ovo* probably disturbs the activation of mechanical stimulus regulated genes which may result in abnormal limb development [3,35]. Mechanosensitive genes influence essential steps of the formation of a proper articular cartilage and consequently have impact on the development of a functional joint [2]. Attempts to identify signaling pathways of mechanotransduction increased in the last decade, although we are still far from the full understanding of these mechanisms of musculoskeletal cells [8,36,37,38]. Our group had reported that intermittent mechanical load caused the activation of PKA and consequent phosphorylation of Sox9 transcription factor, which in turn augmented the matrix production in high density cell cultures [9]. It has been reported that human mesenchymal stem cells can differentiate to chondrocytes by mechanical induction via the activation of Bone Morphogenetic Protein-Tumor Growth Factorß (BMP-TGFß) signaling pathways [39,40]. Additionally, IHH was demonstrated to modify chondrocyte differentiation and proliferation *in vivo* under mechanical load [10] as well as PTHrP was shown as connected to mechanical force-related differentiation [10,41]. PACAP is a small neuropeptide which exerts a protecting effect in several pathological conditions [25,42,43]. In a previous work, we have proven the presence and the chondrostimulatory and chondroprotecting effect of the peptide during oxidative stress in high density chondrofying cell cultures [22]. PACAP also was found to prevent the harmful effects of inflammation on articular cartilage in rheumatoid- and osteoarthritis [26,29,44]. Moreover, there is evidence that this neuropeptide takes role in bone remodelling in response to mechanical load [23,29,45,46].

On the basis of the above data, we hypothesized that PACAP signaling may play role in the mechanotransduction of chondrogenic cells. Previously, we demonstrated that either addition of PACAP 1–38 or application of intermittent mechanical load increased the metachromatic cartilage matrix production in micromass cell cultures [9,22]. In this work we found that the parallel applications of the two interventions, when mechanical load was repeated on the days of final commitment of chondrogenic cells (*i.e.*, days two and three of culturing and PACAP was present continuously) were not additional. This observation may indicate that different intracellular downstream signaling mechanisms can operate upon the two interventions which may interfere to each other. It is worth to note, that administration of PACAP 1–38 or mechanical load on a single day such as day two or day three during the six-day-long culturing period of micromass cell cultures, did not result in any significant alteration of the cartilage formation (data are not shown). Beyond the observed differences in the amount of the metachromatic glycosaminoglycan production, mechanical load, and PACAP exerted different effect on the expression of the main cartilage specific collagens, type II, IX and X. The applied mechanical stimulus seemed to shift the collagen production toward a type of prehypertrophic profile [47], as the presence of type X collagen was increased in the cultures, while addition of PACAP attenuated this effect of mechanical load. The inconsistency of the mRNA data and protein levels of collagen type X may reflect on posttranslational or protein degradation influencing effects of mechanical load rather than transcription modulation in relation to this matrix component. We have detected very similar pattern of the results in mRNA expression and protein levels during PACAP administration and oxidative stress in relation to other ECM proteins such as collagen type II and aggrecan core protein [22]. The elevated presence of collagen type X in the ECM is a key event during chondrocyte hypertrophy leading to calcification of cartilage matrix [48]. It is worthy of note, that a simultaneous significant elevation of the amount of type IX collagen may reflect on certain matrix reorganization upon mechanical load. The presence of collagen type IX determines the architecture of the collagen network in cartilage and its expressional changes have been demonstrated in aging [49]. Addition of PACAP elevated the amount of collagen type II, but reduced the type IX collagen-content in micromass cultures [50]. Strong mechanical load is known to induce the apoptotic program of chondrocytes or mesenchymal stem cells resulting in a shift toward the prehypertrophic phenotype [39,51]. In mature joints, intense mechanical force can result in the reduction of the superficial layer of articulating cartilage and may cause a decrease of the epiphysial growth plate followed by a reduced expression of collagen type II and X [52]. As an important observation, we found that administration of PACAP 1–38 reduced the expression of collagen type X during mechanical stimulation.

This result raises the possibility that PACAP may protect articular cartilage from degenerative diseases in which overload is a well-known etiopathogenetic factor [26,44]. Indeed, when we investigated the expression of PACAP neuropeptide and its receptors upon mechanical load, an increased mRNA expression of PACAP and an elevated protein expression of PAC1 receptor were detected. The protein expressions of VPAC1 and VPAC2, the less effective PACAP receptors, were decreased. PAC1 has been proven as the most feasible receptor responsible for PACAP binding in HD cell cultures [22]. Similar to certain voltage-gated potassium channels [53] which are involved in the regulation of chondrogenesis in HDC, protein expression of PAC1 receptor also showed a peak on day three of culturing, when final commitment of chondrogenic cells occurs in HDC [22]. As the protein expression of PAC1 receptor showed an upregulation during mechanical load we hypothesised that this GPCR receptor is the major component of PACAP regulated signaling pathways in mechanotransduction during embryonic cartilage formation. On the contrary, VPAC1 protein became almost undetectable upon mechanical load, while VPAC2 protein was expressed at a very low level even in control cultures. In the light of these observations contribution of these receptors in mechanosensitivity of chondrogenic cells seems very unlikely. Although the expression of VPAC1 and VPAC2 has been proven in a mesenchymal derived osteoblastic cell line [54] as well as in rat osteosarcoma UMR-106 cells [23] and the involvement of VPAC1 receptor in mechanical induction of osteoblast differentiation has also been demonstrated [46], this function seems tissue specific.

PACAP is known as a general protecting substance in various tissues during different pathological conditions, it exerted neuroprotective [55], nephroprotective effects [25] or reduced the symptoms of acute ileitis [27]. Since mechanical load can be regarded as a physical stress to the chondrogenic cells, one can suppose a mechanoprotective role of PACAP during chondrogenesis. Indeed, this idea was supported by the results obtained during investigating Hedgehog signaling influenced either by PACAP or mechanical load.

Proliferation and hypertrophic program of chondrocytes is under a well-balanced regulation by IHH, PTHrP and SHH signaling cascades, therefore we investigated the involvement of these pathways [56]. PACAP administration diminished the level of SHH and IHH. A similar effect has been detected in motoneuron formation, where the administration of PACAP neuropeptide inhibited the SHH expression [57,58]. It has also been demonstrated that the increase of PKA activity upon engagement of PAC1 receptor with PACAP inhibited the nuclear translocation of Gli1 transcription factor [23,57,58]. In our experiments, mechanical load negatively influenced the protein expression of Gli2 transcription factor, one of the major downstream targets of hedgehog signaling. Moreover, Gli2 has been shown as a primary cilium specific transcription factor influenced by mechanical load [59] and has also been demonstrated to regulate vascularization of cartilage following chondrocyte hypertrophy [60]. It is remarkable, that the protein level of Gli3, which can function either as a transcription activator or repressor in hedgehog signaling [61], was reduced by mechanical activation, while PACAP increased the level of both activator and the shorter, repressor forms of this protein. These findings suggest that mechanical load activates prehypertrophic transformation of differentiating chondrocytes and it can partly be reversed and/or prevented by PACAP addition in chicken micromass cultures.

In summary, our experiments prove the participation of PACAP-signaling in mechanotransduction and suggest a role of PACAP in the regulation of Hedgehog signaling. These findings also imply that this neuropeptide might be applied for therapy of early stages of osteoarthritis.

## 4. Experimental Section

### 4.1. Cell Culturing

Chicken embryos (Ross hybrid) of Hamburger-Hamilton stages 22–24 were used to establish chondrifying primary micromass cell cultures according to Matta *et al.* [31]. Briefly, distal parts of the limb buds of embryos were removed and droplets from cell suspensions of a density of 15 × 10^6^ cell/mL were inoculated onto the center bottom of plastic six-well plates (Eppendorf, Hamburg, Germany). 100 μL droplets of suspension in each well were used to mechanical loading experiments. Day of inoculation is considered as day zero. Colonies were nourished with Dulbecco’s Modified Eagle medium (Sigma-Aldrich, St. Louis, MO, USA), supplemented with 10% fetal calf serum (Gibco, Gaithersburg, MD, USA) and were kept at 37 °C in the presence of 5% CO_2_ and 95% humidity in a CO_2_ incubator. The medium was changed on every second day.

### 4.2. Mechanical Load and PACAP Administration

PACAP 1–38 at 100 nM (stock solution: 100 μM, dissolved in sterile distilled water) applied continuously from day one until the end (day six) of culturing was used as an agonist of PAC1 receptor. PACAP was synthesized as previously described [62]. Micromass cultures grown in six-well plates were subjected to uniaxial cyclic compressive force (approx. 600 Pa, 0.05 Hz) on culturing days two and three for 30 min on both days using a custom-made mechanical stimulator unit (for a detailed description of the bioreactor, please see [9]). This instrument contains pedicles which immerse in the culture medium and could move up and down in the medium in a 2 mm way with the above mentioned frequency, without touching the cell cultures themselves. The exerted mechanical stimulus has two major components, *i.e.*, hydrostatic pressure and fluid shear. Control cultures were grown under identical culture conditions without mechanical stimulation or PACAP addition.

### 4.3. Light Microscopical Morphology

High-density cultures established from 100 μL droplets of limb bud mesenchymal cells of different experimental groups were cultured on the surface of 3 cm diameter round coverglasses (Menzel-Gläser, Menzel GmbH, Braunschweig, Germany) placed into the wells of six-well culture plates. Cell cultures were fixed in a 4:1 mixture of absolute ethanol and 40% formaldehyde on day six of culturing and were stained with 0.1% dimethylmethylene blue (DMMB, Sigma-Aldrich) dissolved in 3% acetic acid, after washing in 3% acetic acid cultures were mounted in gum Arabic. Photomicrographs were taken using an Olympus DP72 camera on a Nikon Eclipse E800 microscope (Nikon Corporation, Tokyo, Japan). For semiquantitaive detection of the amount of metachromatic matrix, we dissolved back toluidine blue (TB; pH 2; Reanal, Budapest, Hungary) from cell cultures on day six. This method provides a good approximation of the amount of formed cartilage as it was described by Matta *et al.* [31]. DMMB and TB metachromatic staining procedures were carried out on separate colonies from the same experiments; DMMB-stained specimens are only shown as visual representations of TB assays. The absorbance of the metachromatic cartilaginous areas was measured in three cultures of each experimental group in three independent experiments. Safranin O staining was carried out in separate colonies. Cell cultures were fixed in a 4:1 mixture of absolute ethanol and 40% formaldehyde on day six of culturing and were stained with 0.1% Safranin O (Sigma-Aldrich) dissolved in distilled water. Cultures were rinsed with 1% acetic acid for 15 s then stained for 5 min. After washing in 1% acetic acid cultures were mounted in gum Arabic.

### 4.4. Measurement of Collagen Production with ^3^H-Proline Labelling

DMEM medium containing 1 μCi/mL ^3^H-proline (diluted from proline [6-^3^H] 20–30 Ci/mmol; 0.74–1.11 TBq/mmol), American Radiolabeled Chemicals, Inc., St. Louis, MO, USA) was added to cell cultures for 24 h on day three after mechanical stress. After washing with PBS, proteins were precipitated with ice-cold 5% trichloroacetic acid, washed with PBS again. Colonies were dried for one week and radioactivity was counted by a Chameleon liquid scintillation counter (Hidex Ltd., Turku, Finland). Measurements were carried out in six samples of each experimental group in four independent experiments. Data were statistically analyzed with F-test.

### 4.5. Preparation of Cell Extracts

Three-day-old cell cultures were washed in physiological NaCl solution and were harvested. After centrifugation, cell pellets were suspended in 100 μL of homogenization RIPA (Radio Immuno Precipitation Assay)-buffer (150 mM sodium chloride; 1.0% NP_4_0, 0.5% sodium deoxycholate; 50 mM Tris, pH 8.0) containing protease inhibitors (Aprotinin (10 µg/mL), 5 mM Benzamidine, Leupeptin (10 µg/mL), Trypsine inhibitor (10 µg/mL), 1 mM PMSF, 5 mM EDTA, 1 mM EGTA, 8 mM Na-Fluoride, 1 mM Na-orthovanadate). Samples were stored at −70 °C. Suspensions were sonicated by pulsing burst for 30 s at 40 A (Cole-Parmer, Vernon Hills, IL, USA). For Western blotting, total cell lysates were used. Samples for SDS-PAGE were prepared by the addition of Laemmli electrophoresis sample buffer (4% SDS, 10% 2-mercaptoehtanol, 20% glycerol, 0.004% bromophenol blue, 0.125 M Tris HCl pH 6.8) to cell lysates to set equal protein concentration of samples, and boiled for 10 min. For RT-PCR analysis cartilage colonies were washed three times with RNAse-free physiological sodium chloride then the cultures were stored at −70 °C.

### 4.6. RT-PCR Analysis

Cell cultures were dissolved in Trizol (Applied Biosystems, Foster City, CA, USA), 20% RNase free chloroform was added and the samples were centrifuged at 4 °C on 10,000 rpm for 15 min. Samples were incubated in 500 µL of RNase free izopropanol in −20 °C for 1 h then total RNA was harvested in RNase free water and stored at −20 °C. The assay mixture for reverse transcriptase reaction contained 2 µg RNA, 0.112 µM oligo(dT), 0.5 mM dNTP, 200 units High Capacity RT (Applied Bio-Systems, Foster City, CA, USA) in 1× RT buffer. For the sequences of primer pairs and further details of polymerase chain reactions, see Table 1. Amplifications were performed in a thermal cycler (Labnet MultiGene™ 96-well Gradient Thermal Cycler; Labnet International, Edison, NJ, USA) in a final volume of 11 μL (containing 0.5 μL forward and reverse primers (0.4 μM), 0.25 μL dNTP (200 μM), and five units of Promega GoTaq^®^ DNA polymerase in 1× reaction buffer) as follows: 95 °C, 2 min, followed by 35 cycles (denaturation, 94 °C, 1 min; annealing at optimized temperatures as given in Table 1 for 1 min; extension, 72 °C, 90 s) and then 72 °C, 10 min. PCR products were analyzed by electrophoresis in 1.2% agarose gel containing ethidium bromide. GAPDH was used as an internal control. Optical density of signals was measured by using ImageJ 1.40g freeware (http://rsbweb.nih.gov/ij/) and results were normalized to the optical density of untreated control cultures.

### 4.7. Western Blot Analysis

About 10 µg of protein was separated by 7.5% SDS-PAGE gel for detection of PAC1, VPAC1, VPAC2, Actin, Collagen type II, Collagen type IX, Collagen type X, PTHrP, SHH, IHH, Smoothened (Smo), Gli1, Gli2, and Gli3. Proteins were transferred electrophoretically to nitrocellulose membranes. After blocking with 5% non-fat dry milk in phosphate buffered saline (PBST) with 0.1% Tween 20, membranes were washed and exposed to the primary antibodies overnight at 4 °C in the dilution as given in Table 2. After washing for 30 min in PBST, membranes were incubated with anti-rabbit IgG (Bio-Rad Laboratories, Hercules, CA, USA) in 1:1500 or anti-mouse IgG (Bio-Rad Laboratories) in 1:1500 dilution. Signals were detected by enhanced chemiluminescence (Pierce™, Thermo Fisher Scientific Inc., Waltham, MA, USA) according to the instructions of the manufacturer. Signals were developed with gel documentary system (Fluorchem E, ProteinSimple, San Jose, CA, USA). Optical density of Western blot signals was measured by using ImageJ 1.40g freeware and results were normalized to that of untreated control cultures.

**Table 1:** Nucleotide sequences, amplification sites, GenBank accession numbers, amplimer sizes and PCR reaction conditions for each primer pair are shown.

Gene	Primer	Nucleotide Sequence (5′→3′)	GenBank ID	Annealing Temperature	Amplimer Size (bp)
Collagen II (*Col2a1*)	sense	GGA CCC AAA GGA CAG ACG G (1191–1210)	NM_204426	59 °C	401
	antisense	TCG CCA GGA GCA CCA GTT (1573–1591)			
Collagen IX (*Col9a1*)	sense	GGG ACAA GAG GAA TAA ACG (1732–1750)	NM_001100911.1	52 °C	163
	antisense	CTG GTA AAC CTG GCA ATC (1877–1894)			
Collagen X (*Colxa1*)	sense	TCT GGG ATG CCG CTT GTC (1681–1698)	NM_009925.4	56 °C	261
	antisense	CGT AGG CGT GCC GTT CTT (1924–1941)			
PACAP (*ADCYAP1*)	sense	CTT CGC ACT ACG AGC AGG (156–163)	NM_001001291	52.5 °C	198
	antisense	TTG ACA GCC ATT TGT TTC C (335–363)			
PAC1 (*ADCYAP1R1*)	sense	GTC AGA CAA CCA GGA TTA C (435–453)	NM_001098606	49 °C	141
	antisense	TGG ATA AAG TTC CGA GTG (559–575)			
VPAC1 (*VIPR1*)	sense	GTT CTA TGG CAC GGT CAA (376–393)	NM_001097523	52 °C	216
	antisense	AGC AAT GTT CGG GTT CTC (573–590)			
VPAC2 (*VIPR2*)	sense	TCG GAA CTA CAT CCA TCT (477–497)	NM_001014970	48 °C	177
	antisense	TTT GCC ATA ACA CCA TAC (636–653)			
SHH (*Shh*)	sense	TCA GTG GCA GCG AAA TCA (787–804)	NM_204821.1	56 °C	168
	antisense	CAT CCG GTC GAG GAA GGT (937–954)			
IHH (*Ihh*)	sense	TCG CCT ACA AGC AGT TCA GCC (455–475)	NM_204957.1	60 °C	155
	antisense	GCC GGT GTT CTC CTC GTC CT (590–609)			
PTHrP (*Pthlh*)	sense	TAC GGA AGA TCA GTA GAG G (155–173)	NM_001174106.1	46 °C	191
	antisense	GTA GCA GGC TTA GGG TTA (328–345)			
Smoothened (*SMO*)	sense	TCT GCT TCG TGG GTT ACA AG (843–862)	XM_414970.4	56 °C	350
	antisense	TGG GAT GGG TTT ATT GGT CT (1173–1192)			
Gli1 (*Gli1*)	sense	CTC ACC CAC CCA GCA TCA G (2604–2622)	XM_004950861.1	58 °C	244
	antisense	AAT CCC TCC TCC ATC TCC CT (2828–2847)			
Gli2 (*Gli2*)	sense	TTG CTC CAA GGC TTA CTC (1553–1570)	M_001271901.1	50 °C	174
	antisense	TTA CAG ACA TAG GGT TTC TCA T (1705–1726)			
Gli3 (*Gli3*)	sense	TCA CCC GTA CAT TAA CCC (579–596)	NM_001271903.1	52 °C	270
	antisense	CTT GGA CTC GGA AAC CTG (831–848)			
GAPDH (*GAPDH*)	sense	GAG AAC GGG AAA CTT GTC AT (238–258)	NM_204305	54 °C	556
	antisense	GGC AGG TCA GGT CAA CAA (775–793)			

**Table 2:** Tables of antibodies used in the experiments.

Antibody	Host Animal	Dilution	Distributor and Cat No.
Anti-PAC1	rabbit, polyclonal,	1:600	Sigma-Aldrich, St. Louis, MO, USA; P8872
Anti-VPAC1	rabbit, polyclonal,	1:800	Alomone Labs., Jerusalem, Israel; AVR-001
Anti-VPAC2	rabbit, polyclonal,	1:600	Abcam, Camridge, UK; ab28624
Anti-Coll. II.	rabbit, polyclonal,	1:100	Novus Biologicals, Littleton, CO, USA; NB600-844
Anti-Coll. IX.	rabbit, polyclonal,	1:800	Abcam, Camridge, UK; ab134568
Anti-Coll. X.	rabbit, polyclonal,	1:800	Sigma-Aldrich, St. Louis, MO, USA; C7974
Anti-SHH	rabbit, polyclonal,	1:600	Cell Signaling, Danvers, MA, USA; C9C5
Anti-IHH	rabbit, polyclonal,	1:600	Millipore, Billerica, MA, USA; MABF23
Anti-PTHrP	mouse, monoclonal, #677939	1:300	R&D Systems, Minneapolis, MN, USA; MAB6734
Anti-Smoothened	mouse, monoclonal, #2D10	1:500	Sigma-Aldrich, St. Louis, MO, USA; SAB1412475
Anti-Gli1	rabbit, polyclonal,	1:600	Cell Signaling, Danvers, MA, USA; V812
Anti-Gli2	rabbit, polyclonal,	1:500	Sigma-Aldrich, St. Louis, MO, USA; SAB2900411
Anti-Gli3	rabbit, polyclonal,	1:500	Sigma-Aldrich, St. Louis, MO, USA; HAP005534
Anti-Actin	mouse, monoclonal, #AC-15	1:10,000	Sigma-Aldrich, St. Louis, MO, USA; A5441

### 4.8. Statistical Analysis

All data are representative of at least three independent experiments. Averages are expressed as mean ± SEM (standard error of the mean). Statistical analysis was performed by unpaired Student’s *t*-test. Threshold for statistically significant differences as compared to control cultures was set at * *p* < 0.05). Statistical analysis and comparison of Western blot and PCR results can be seen in Figure 2C,E, Figure 3B,D, and Figure 4B,D.

## Abbreviations

cAMP: cyclic adenosine monophosphate
CREB: cAMP response element-binding protein
DMMB: dimethylmethylene blue
ECM: extracellular matrix
HH: hedgehog
IHH: Indian hedgehog
MAPK: mitogen-activated protein kinase
NMDA: *N*-Methyl-d-aspartate
PAC1: pituitary adenylate cyclase-activating polypeptide type I receptor
PACAP: pituitary adenylate cyclase polypeptide
PKA: protein kinase A
PTHrP: parathyroid hormone related peptide
Runx2: Runt-related transcription factor 2
SHH: Sonic hedgehog
TB: toluidine blue
TGFβ: transforming growth factor-β
TRPV: transient receptor potential channels for vanilloid
VIP: vasoactive intestinal peptide
VPAC: vasoactive intestinal peptide receptor
